# Study on the Mechanism of mTOR-Mediated Autophagy during Electroacupuncture Pretreatment against Cerebral Ischemic Injury

**DOI:** 10.1155/2016/9121597

**Published:** 2016-07-28

**Authors:** Zhou-Quan Wu, Su-yang Cui, Liang Zhu, Zhi-qing Zou

**Affiliations:** ^1^Department of Anesthesiology, The Affiliated Hospital of Nanjing Medical University, Changzhou No. 2 People's Hospital, Changzhou 213000, China; ^2^The First Clinical College, Nanjing University of Chinese Medicine, Nanjing 210029, China

## Abstract

This study is aimed at investigating the association between the electroacupuncture (EA) pretreatment-induced protective effect against early cerebral ischemic injury and autophagy. EA pretreatment can protect cerebral ischemic and reperfusion injuries, but whether the attenuation of early cerebral ischemic injury by EA pretreatment was associated with autophagy is not yet clear. This study used the middle cerebral artery occlusion model to monitor the process of ischemic injury. For rats in the EA pretreatment group, EA pretreatment was conducted at Baihui acupoint before ischemia for 30 min for 5 consecutive days. The results suggested that EA pretreatment significantly increased the expression of autophagy in the cerebral cortical area on the ischemic side of rats. But the EA pretreatment-induced protective effects on the brain could be reversed by the specific inhibitor 3-methyladenine of autophagy. Additionally, the Pearson correlation analysis indicated that the impact of EA pretreatment on p-mTOR (2481) was negatively correlated with its impact on autophagy. In conclusion, the mechanism of EA pretreatment at Baihui acupoint against cerebral ischemic injury is mainly associated with the upregulation of autophagy expression, and its regulation of autophagy may depend on mTOR-mediated signaling pathways.

## 1. Introduction

Ischemic cerebrovascular disease is one of the most serious diseases that threaten people's lives. Studies have discovered that electroacupuncture (EA) pretreatment has a protective effect on cerebral ischemic injury, and the mechanism is commonly thought to be a multitarget, multilevel, and multichannel integrated system. For example, the stability of blood-brain barrier is maintained by decreasing the activity of matrix metalloproteinase-9 [1–3]; the apoptosis of cells is inhibited by regulating adenosine receptor [4], opioid receptor [5], cannabinoid receptor [6–9], *N*-methyl-D-aspartate receptors [10], and intracellular activity. Different to the studies, our recent study further found that EA could alleviate cerebral reperfusion injury by inhibiting autophagy [11].

Autophagy is an evolutionarily conserved pathway that involves the sequestration and delivery of cytoplasmic materials to the lysosomes, where proteins are degraded and recycled. Recent studies hold that autophagy is a double-edged sword in cell survival. In some conditions, autophagy activation may promote cell survival by digesting the misfolded protein [12, 13]. In other conditions, autophagy activation could exacerbate cerebral cell apoptosis [14, 15]. Thus, this study aimed to further investigate the association between the EA pretreatment-induced protective effect against early cerebral ischemic injury and autophagy.

## 2. Experimental Methods

### 2.1. Study Population and Grouping

Sprague-Dawley (SD) male rats weighing 280–320 g were included in the study. The middle cerebral artery occlusion (MCAO) model was adopted as a focal cerebral ischemia model. Rats were randomly divided into the following five groups: sham, ischemia (I), EA pretreatment (EA+I), autophagic specific inhibitor 3-methyladenine (3-MA) (3-MA+EA+I), and vehicle (Veh+EA+I), with 23 rats in each group. For rats in the sham group, the right common carotid was exposed under anesthesia without embolization; pentobarbital sodium was given as preoperative anesthesia without EA pretreatment. For rats in the ischemia group, thread embolization was conducted for 2 h after the common carotid artery was exposed under anesthesia; pentobarbital sodium was given as preoperative anesthesia without EA pretreatment. For rats in the EA pretreatment group, treatment was almost the same as that for rats in the ischemia group, except that EA pretreatment was conducted after anesthesia. For rats in the 3-MA group, treatment was the same as that for rats in the EA pretreatment group; additionally, intracerebroventricular injection of 20 *μ*L 3-MA (400 nmol) was given 30 min before ischemia. For rats in the vehicle group, treatment was the same as that for rats in the 3-MA group, except that 3-MA was replaced with dimethyl sulfoxide.

### 2.2. Preparation of Middle Cerebral Artery Models [16]

Rats were subjected thread embolization in the middle cerebral artery for 2 h. Cerebral blood flow (CBF) through the middle cerebral artery was measured by laser Doppler flowmetry. Animals with less than 80% reduction in CBF in the core of the middle cerebral artery area were excluded from this study.

### 2.3. Electroacupuncture Pretreatment

EA pretreatment was performed at the acupoint “Baihui (GV20).” The acupoint “Baihui” is located at the intersection of sagittal midline and the joint of line between the two ears [17]. Animals were anesthetized and stimulated with the density-sparse wave of 2/15 Hz and an intensity of 1 mA for 30 min/day for five consecutive days prior to focal cerebral ischemia using the Hwato Electronic Acupuncture Treatment Instrument (Model No. SDZ-V, Suzhou Medical Appliances Co., Ltd., Suzhou, China) [18].

### 2.4. TUNEL Assay

The terminal deoxynucleotidyl transferase dUTP nick end labeling (TUNEL) assay was performed based on manufacturer's instructions. Brain sections were stained with diaminobenzidine and counterstained with hematoxylin. Gradient alcohol was then applied for dehydration, and finally the sections were cleared with xylene and mounted with neutral resin. The reaction mixture was replaced with phosphate-buffered saline (PBS) in the negative control. The nucleus was blue and the apoptotic nucleus was brownish-black or brown. A slice from each rat was observed, and five fields (magnification, 400x) were counted on each slice. The percentage of positive apoptotic nuclei was calculated from the total number of cells per field, the mean value of which was regarded as the apoptotic index of cerebral neurons.

### 2.5. Transmission Electron Microscopic Examination [19]

Two hours after ischemia, rats were perfused with precooled PBS (pH 7.4) followed by PBS containing 4% paraformaldehyde and 0.25% glutaraldehyde after anesthetization. The brains were then taken out and kept overnight in 2% paraformaldehyde and 2.5% glutaraldehyde in 0.1 M PBS (pH 7.4). On the next day, the brains were cut into 50 mm thick slices by a vibratome, and the parietal lobe cortex was selected for analysis. The selected slices were treated by postfixation in 1% osmium tetroxide for 1 h, followed by dehydration in graded ethanol. Finally, samples were embedded in epoxy resin. Polymerization was performed at 80°C for 24 h, and blocks were cut on a Reichert ultramicrotome into ultrathin sections (60–70 nm), which were poststained with uranyl acetate and lead citrate, and observed under a Hitachi 7100 electron microscopy (Nikon, Tokyo, Japan). Ten fields of each rat (three rats in each group) were examined by applying the protocol described earlier to conduct quantitative analysis on the number of autophagosomes.

### 2.6. Western Blotting [20]

Two hours after ischemia, the parietal lobe cortex of the right middle cerebral artery territory and the corresponding area of sham-operated rats were homogenized. A lysis buffer [10 mM Tris-HCl, pH 7.4, 150 mM NaCl, 1% Triton-100, 0.1% sodium dodecyl sulfate (SDS), 5 mM EDTA, 1 mM phenylmethyl sulfonate fluoride, 0.28 U/mL aprotinin, 50 *μ*g/mL leupeptin, 1 mM benzamidine, and 7 *μ*g/mL pepstatin A] was applied for protein extraction, and protein concentration was determined by spectrophotometer (UV-2540, Shimadzh Corp., Kyoto, Japan). A 60 *μ*g aliquot of proteins from each sample was extracted by applying 10% SDS-polyacrylamide gel electrophoresis and transferred to a nitrocellulose membrane, which was then incubated with the specific antibodies against LC3 (#4108; 1 : 1,000; Cell Signaling Technology, Inc., Danvers, MA,USA), p-mTOR (#2971, Cell Signaling Technology, Inc., Danvers, MA, USA), and mTOR (#2972, Cell Signaling Technology, Inc., Danvers, MA, USA) at 4°C overnight. Afterward, the membranes were incubated with a horseradish peroxidase-conjugated secondary antibody at room temperature for 1 h. An enhanced chemiluminescent autoradiography was adopted to detect immunoreactivity based on manufacturer's instructions. Membranes were reprobed with  *β*-actin after stripping and finally used for determining protein expression with Sigma Scan (scanPDF15; Sigma-Aldrich) and normalized to the loading control.

### 2.7. Evaluation of Infarct Volume and Brain Water Content [1]

Two hours after ischemia, the rats were killed and the brains were dissected and sliced for 2% 2,3,5-triphenyltetrazolium chloride (TTC). Infarct volume = (total wet weight − red weight)/total wet weight × 100%; water content = (wet weight − dried weight)/wet weight × 100%.

### 2.8. Statistical Analysis

The SPSS 15.0 (SPSS, Inc., Chicago, IL, USA) statistical software was adopted for statistical analysis. Count data was analyzed by univariate analysis, and pairwise comparisons were conducted by independent *t* test. The Pearson correlation analysis was conducted for p-mTOR and LC3-II/LC3-I ratio after EA pretreatment.

## 3. Results

### 3.1. Brain Cell Apoptosis for Rats in Each Group

**Figure 1:**
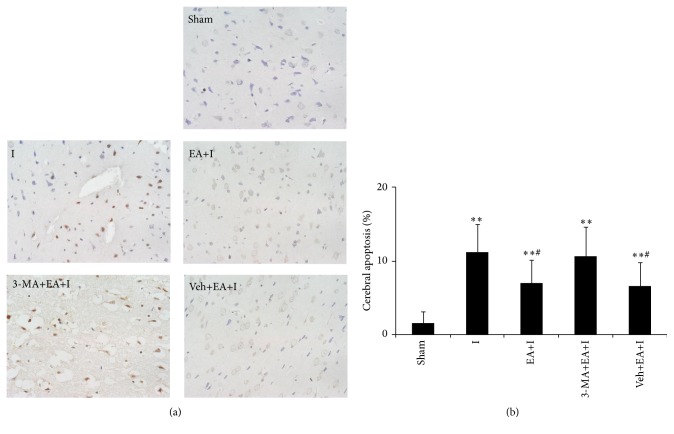
Rats were killed 2 h after cerebral ischemia and processed for TUNEL assay. (a) The brown nucleus represents the occurrence of apoptosis (magnification, ×400) in the sham, I, EA+I, 3-MA+EA+I, and Veh+EA+I group; (b) quantitative analysis for the percentage of cerebral apoptosis. 10 fields for each rat were examined; *n* = 5 for each group. Bar represents mean ± SD; ^*∗∗*^ *P* < 0.01 compared with the sham group; ^#^ *P* < 0.05 compared with the ischemia group.

Compared with rats in the sham group, cerebral cortex apoptotic cells for rats in I, EA+I, 3-MA+EA+I, and Veh+EA+I groups increased (*P* < 0.01). Compared with rats in the I group, apoptotic cells for rats in EA+I and Veh+EA+I groups significantly decreased (*P* < 0.05), but cerebral apoptotic cells for rats in the 3-MA+EA+I group had no statistical difference (*P* > 0.05). Results are shown in Figure 1.

### 3.2. Changes in Areas of Brain Edema and Cerebral Infarction for Rats in Every Group

**Figure 2:**
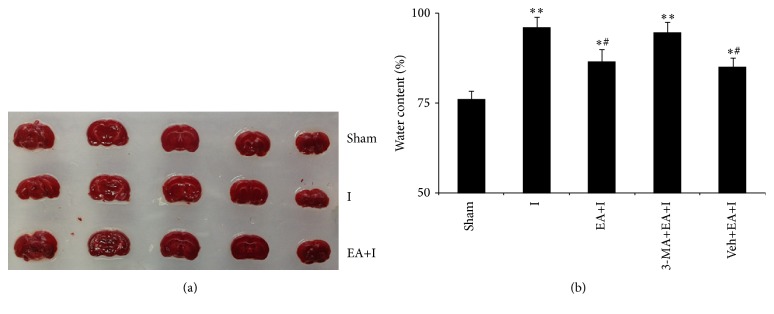
Rats were killed 2 h after cerebral ischemia. (a) Representative infarcts with TTC staining for different groups are shown. (b) Measurement of brain edema from different groups 2 h after ischemia. *n* = 8 for each group. ^*∗*^ *P* < 0.05 and ^*∗∗*^ *P* < 0.01, compared with the sham group; ^#^ *P* < 0.05, compared with the ischemia group.

Compared with rats in the sham group, brain water content increased for rats in I, EA+I, 3-MA+EA+I, and Veh+EA+I groups, while brain water content for rats in EA+I and Veh+EA+I groups significantly decreased (*P* < 0.05) compared with rats in the I group. No significant difference was observed for brain water content for rats in the EA+I2h+3-MA group (*P* > 0.05). Moreover, no obvious statistical difference was observed in cerebral infarction for rats in sham, I, and EA+I groups (*P* > 0.05), as shown in Figure 2.

### 3.3. Changes in the LC3-II/LC3-I Ratio for Rats in Every Group

**Figure 3:**
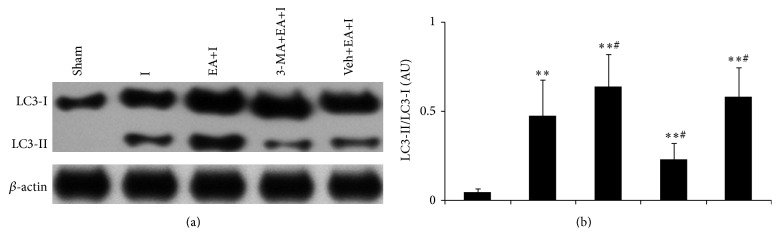
Extracts from the cerebral cortex were separated for immunoblotting. (a) Representative blots for LC3 are shown in different groups. Levels of  *β*-actin protein were used as loading control. (b) Bar represents mean ± SD from 5 rats in each group. ^*∗∗*^ *P* < 0.01 compared with the sham group; ^#^ *P* < 0.05 compared with I group.

Compared with rats in the sham group, cerebral cortex LC3-II protein expression increased for rats in I, EA+I, 3-MA+EA+I, and Veh+EA+I groups (*P* < 0.05 or *P* < 0.01). LC3-II protein expression also increased for rats in EA+I and Veh+EA+I groups compared with rats in the I group, while LC3-II protein expression decreased for rats in the 3-MA+EA+I group (*P* < 0.05). No significant statistical difference was observed for LC3-I protein expression of every group (*P* > 0.05), and thus the trend for LC3-II/LC3-I ratio was the same as that of LC3-II protein, as shown in Figure 3.

### 3.4. Changes in Ultrastructure under an Electron Microscope for Rats in Every Group

**Figure 4:**
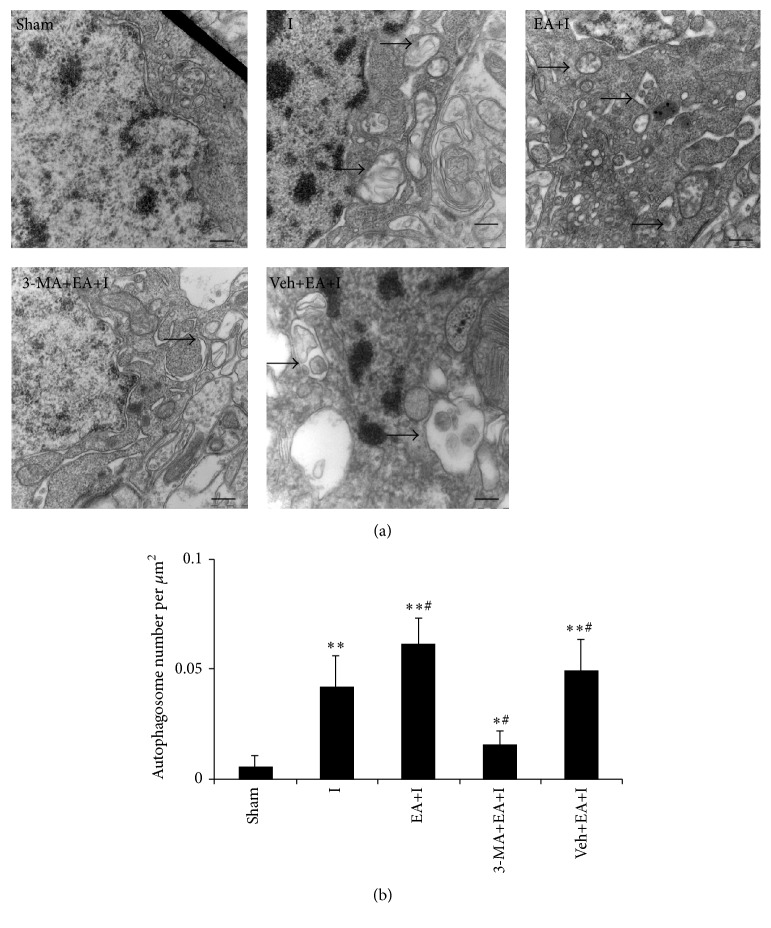
Rats were killed 2 h after ischemia and processed for electron microscopic examination. (a) Representative images are shown for each group. Scale bar: 0.5 *μ*m. Arrows indicate autophagosome; (b) quantitative analysis for the number of autophagosome in the sham, I, EA+I, 3-MA+EA+I, and Veh+EA+I group. Bar represents mean ± SD; ^*∗*^ *P* < 0.05 and ^*∗∗*^ *P* < 0.01, compared with the sham group; ^#^ *P* < 0.05, compared with the ischemia group.

Compared with rats in the sham group, a cupular separation film, formed by membrane shedding of an autophagosome from sources such as Golgi apparatus, in the nonribosomal region of the rough endoplasmic reticulum for rats in every group, was observed to be wrapped around degradation matter (part of the cytoplasm, organelles, and proteins that needed degradation inside cells). However, the increase of autophagosomes was more significant for rats in EA+I and Veh+EA+I groups (*P* < 0.05) than for rats in the I group. Autophagosomes for rats in the 3-MA+EA+I group obviously decreased (*P* < 0.05), as shown in Figure 4.

### 3.5. Changes in Cortex p-mTOR Expression for Rats in Every Group

**Figure 5:**
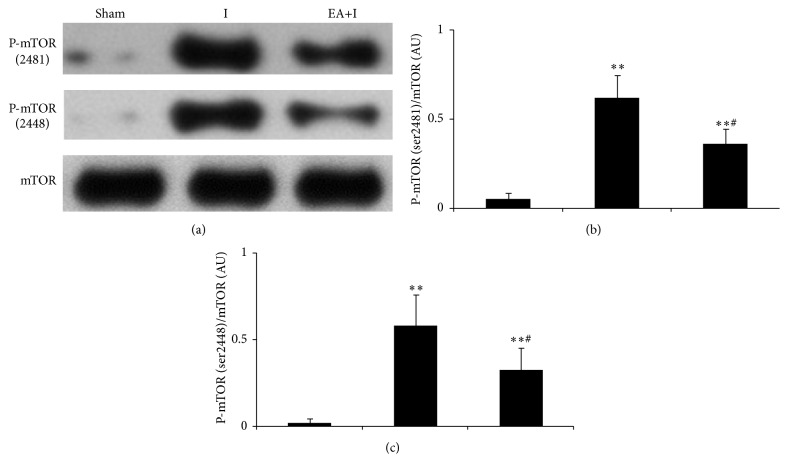
Extracts from the cerebral cortex were separated for immunoblotting. (a) Representative blots for p-mTOR and mTOR are shown in different group. (b) Bar represents the ratio of p-mTOR (2481)/mTOR. (c) Bar represents the ratio of p-mTOR (2448)/mTOR; each group, *n* = 5. ^*∗∗*^ *P* < 0.01 compared with the sham group; ^#^ *P* < 0.05 compared with the ischemia group.

Compared with the sham group, the phosphorylation level of two serine sites in cortex mTOR increased in all groups but decreased in the EA+I group compared with that in the I group (*P* < 0.05), as shown in Figure 5.

### 3.6. Results of Pearson Correlation Analysis

It was found that p-mTOR (2481) and LC3-II/LC3-I ratio were negatively correlated after EA treatment (*r* = −0.89, *P* = 0.04); P-mTOR (2448) and LC3-II/LC3-I ratio were not correlated (*r* = −0.34, *P* = 0.59).

## 4. Discussion

EA is a technological product when combining the traditional acupuncture and modern electrotherapy. It is well known to all that the acupoint, skill, and stimulus intensity are the base of acupuncture. Likewise, the choice of acupoint and electric stimulation parameters (frequency, pulse, width, current intensity, and duration) are important for EA [21]. Based on meridian theory, the “Baihui” acupoint (one of the acupoints of Du meridian) was selected for use in the present study as it receives projections from the motor and sensory cortex, as well as from the anterior cerebral artery. In addition, many studies have reported that the different stimulation parameters can exert different effect on body. According to the reports of Li et al. [22], the appropriate electrical stimulation parameters of EA pretreatment to induce cerebral ischemic tolerance in rats are density-sparse wave of 2/15 Hz and an intensity of 1 mA for 30 min/day over five consecutive days. Moreover, our previous study that “Baihui acupoint (GV20)”-stimulation with the same parameters induce significant cerebral ischemia/reperfusion tolerance [11].

Autophagy is a process in which an autophagosome is formed through monolayer or bilayer wrapping of intracellular substrates that are to be degraded, and its detection usually depends on morphological observations or iconic protein on autophagosome membranes. For morphological observations, transmission electron microscopy is applied for real-time observation on the ultrastructure of autophagosome [23]. The iconic protein on autophagosome membranes mainly includes microtubule-associated protein 1 light chain 3 (LC3). It is a homologue of yeast autophagic gene (*Apg7/Apg8*) in mammals, and LC3 exists in every stage of autophagy in the form of LC3-PE, by combining with phosphatidylethanolamine (PE), namely, cephaline [24]. When an autophagosome is formed, cytosolic LC3 (namely, LC3-I) transforms into a membrane (namely, LC3-II) [25]. Autophagic changes at every stage can be well observed, and electron microscopy and LC3-II/LC3-I ratio were mainly selected as detection indicators in this study.

To better observe the association between EA and early cerebral ischemia, pretests were conducted to investigate changes in cerebral infarction area, apoptosis of brain cells, and brain water content in SD rats at 1 h, 2 h, and 4 h after ischemia. As a result, it was observed that after 2 h of cerebral ischemia for rats, cerebral cortex number of apoptotic cells and brain water content significantly increased, but no obvious infarction zone was observed by triphenyl tetrazolium chloride staining (data not show). Observations indicated that manifestation at the points of time complied more with early cerebral ischemia. Thus, this study mainly investigated the protection mechanism of EA treatment for ischemia at 2 h. Based on the results, EA pretreatment had a protective function for early cerebral ischemia, mainly manifested as the decrease of apoptosis of brain cells and brain edema. Moreover, EA pretreatment significantly increased the expression of autophagy. It could be observed through electron microscopy that the number of autophagosomes in the cerebral cortex of rats after EA pretreatment obviously increased, and the autophagic iconic membrane protein LC3-II/LC3-I ratio also increased. However, autophagic specific inhibitor 3-MA [mainly for the inhibition of the transformation process of LC3-I to LC3-II [26]] could significantly weaken the EA pretreatment-induced protective effects on the brain along with the decrease of autophagy, which indicated that, at early cerebral ischemia, the EA pretreatment-induced cerebral protective mechanism was mainly associated with the upregulation of autophagic expression. Of course, there exist a debatable problem where 3-MA seems to counteract completely the protective effect of EA (Figures 1 and 2). The most possible explanation is that 3-MA can inhibit most part of autophagy (induced by EA pretreatment and ischemia insult [27]). Therefore, it not only weakened the protective effect of EA mediating autophagy but also inhibited the neuroprotection by activation of autophagy following the ischemia insult. Moreover, autophagy have some association with the other targets of EA, such as the matrix metalloproteinase-9 [28], adenosine receptor [29], and cannabinoid receptor [30].

To study the internal mechanism of the increase of autophagic expression by EA pretreatment during early cerebral ischemia, the relationship between EA pretreatment and mTOR was investigated in this study. The autophagic regulatory pathways can be divided into mTOR regulatory pathway and mTOR independent regulatory pathway [31]. mTOR is an atypical serine/threonine protein kinase and is a family membrane of phosphatidylinositol kinase-related kinase protein [32]. Its activation mainly depends on the phosphorylation of 2448 and (or) 2481 serine loci, and the activation of mTOR can inhibit autophagic expression. Based on experimental results, EA pretreatment significantly suppressed mTOR activity during cerebral ischemia, mainly manifested as the decrease of the phosphorylation of 2448 and (or) 2481 serine loci of mTOR. Results from correlation analysis suggested that p-mTOR (2481) and LC3-II/LC3-I ratio were negatively correlated after EA pretreatment. The aforementioned discoveries indicated two points: first, the brain cell autophagy that occurred during cerebral ischemia was mainly mTOR-dependent, and, second, EA pretreatment at Baihui acupoint may further regulate autophagic expression through the mTOR pathway.

In summary, this study proved that EA pretreatment at Baihui acupoint had a protective function on the brain by upregulation of autophagy during early cerebral ischemia, and its regulation of autophagy may depend on the mTOR signal pathway. Although the conclusion is contrary to that of our previous study, it is not contradictory. In the previous study, the stage is cerebral reperfusion. Therefore, the conclusions in this study replenished the conclusion in the previous study and further testified that EA have the function of bidirectional regulation. Of course, there were some limitations: this study only proved the correlation between p-mTOR and autophagy, which could be more persuasive if it was further validated by specific inhibitors of p-mTOR. These limitations are expected to be improved in further studies.
